# Antiviral Lectins from Red and Blue-Green Algae Show Potent *In Vitro* and *In Vivo* Activity against Hepatitis C Virus

**DOI:** 10.1371/journal.pone.0064449

**Published:** 2013-05-21

**Authors:** Yutaka Takebe, Carrie J. Saucedo, Garry Lund, Rie Uenishi, Saiki Hase, Takayo Tsuchiura, Norman Kneteman, Koreen Ramessar, D. Lorne J. Tyrrell, Masayuki Shirakura, Takaji Wakita, James B. McMahon, Barry R. O'Keefe

**Affiliations:** 1 AIDS Research Center, National Institute of Infectious Diseases, Tokyo, Japan; 2 Molecular Targets Laboratory, SAIC-Frederick, Frederick, Maryland, United States of America; 3 KMT Hepatech, Edmonton, Alberta, Canada; 4 Molecular Targets Laboratory, Center for Cancer Research, National Cancer Institute, Frederick, Maryland, United States of America; 5 Virology II, National Institute of Infectious Diseases, Tokyo, Japan; University of California Merced, United States of America

## Abstract

Hepatitis C virus (HCV) infection is a significant public health problem with over 170,000,000 chronic carriers and infection rates increasing worldwide. Chronic HCV infection is one of the leading causes of hepatocellular carcinoma which was estimated to result in ∼10,000 deaths in the United States in the year 2011. Current treatment options for HCV infection are limited to PEG-ylated interferon alpha (IFN-α), the nucleoside ribavirin and the recently approved HCV protease inhibitors telaprevir and boceprevir. Although showing significantly improved efficacy over the previous therapies, treatment with protease inhibitors has been shown to result in the rapid emergence of drug-resistant virus. Here we report the activity of two proteins, originally isolated from natural product extracts, which demonstrate low or sub-nanomolar *in vitro* activity against both genotype I and genotype II HCV. These proteins inhibit viral infectivity, binding to the HCV envelope glycoproteins E1 and E2 and block viral entry into human hepatocytes. In addition, we demonstrate that the most potent of these agents, the protein griffithsin, is readily bioavailable after subcutaneous injection and shows significant *in vivo* efficacy in reducing HCV viral titers in a mouse model system with engrafted human hepatocytes. These results indicate that HCV viral entry inhibitors can be an effective component of anti-HCV therapy and that these proteins should be studied further for their therapeutic potential.

## Introduction

The unique genus Hepacivirus contains only one species, commonly referred to as hepatitis C virus (HCV) [1]. Human infection with HCV is generally achieved *via* blood exchange, resulting in a chronic infection of the liver and often, primary hepatocellular carcinoma [2]. There is no vaccine available for HCV and current therapeutic regimens rely on IFN-α and ribavirin; now in combination with the recently approved HCV NS3/4A protease inhibitors (boceprivir, Merck and telaprevir, Vertex/Gilead) [3]. These new agents substantially improve on the previous treatment regimen which only was effective in ∼50% of patients [4]. Unfortunately, due to HCV's poor replicative fidelity, it often becomes resistant to targeted drugs [5]. Therefore, despite the new agents now becoming available, there is a continuing need for additional agents to combat HCV.

Fortunately, the virus presents several unique targets. These include the NS3/4A protease, the NS5b polymerase, the multifunctional NS5a protein, and the surface glycoproteins E1 and E2. Effort is also being put into the development of immunomodulators that affect toll-like receptors or inhibit cyclophilin [6]. Numerous agents that target the NS3/4a protease are currently in clinical trials [7]. Both nucleoside and non-nucleoside inhibitors of NS5b are also in Phase II clinical trials, although several previous trials have been stopped due to toxicity [6]. Clinical studies have also been reported for an NS5a inhibitor, BMS-790052, where response rates were >83% [8]. Though significant pharmaceutical development efforts are addressing the enzyme targets within the HCV genome, the only viral entry inhibitors targeting the surface envelope glycoproteins E1 and E2 previously in clinical trials were neutralizing antibodies [9], [10].

The HCV envelope glycoproteins E1 and E2 are responsible for viral attachment and entry [11]. E1 and E2 have been reported to contain from 5–11 potential N-linked glycosylation sites [12]. The binding of E2 to cell-surface proteins including claudin-1 [13], CD81 and SR-B1 [14] is required for efficient cell entry by HCV. Glycosylation of E2 has been reported to be critical for viral entry [15], E2 expression and folding [16] and modulating response to anti-HCV antibodies [17]. Due to these activities, inhibition of E2 function is an attractive target for therapeutic development.

Viral entry of HCV offers several specific cellular targets in addition to the viral envelope glycoprotein E2. Antibodies to all of these targets have inhibited HCV entry to some extent [18]. In addition, agents, which work by less defined mechanisms, such as silymarin [19] and the lamiridosins [20] also inhibit HCV entry independent of any reduction in E2-coreceptor binding. Inhibition of E2 binding by agents other than antibodies has recently been reported for the lectin cyanovirin-N (CV-N), which inhibited HCV entry at nanomolar concentrations but was never tested for *in vivo* activity [21].

**Figure 1:**
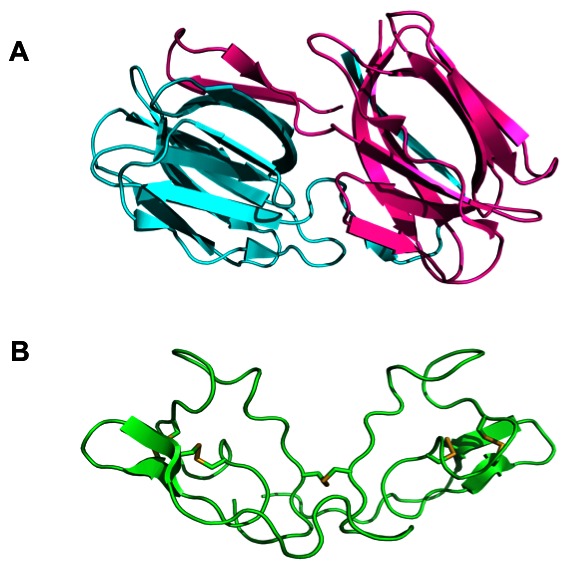
Ribbon diagrams of the three dimensional structures of the lectins griffithsin and scytovirin. (A) griffithsin (GRFT), a 25 kDa-protein (a domain-swapped homodimmer of 12.7-kDa subunit) was originally isolated from the red algae *Griffithsia* sp.; (B) Scytovirin, a 9.7-kDa protein, was originally isolated from the blue-green algae *Scytonema varium*.

Here we report the activity of the proteins griffithsin (GRFT) and scytovirin (SVN) against HCV. GRFT and SVN were originally isolated from red [22] and blue-green algae [23], respectively. Both proteins have unique structural characteristics, which enable them to simultaneously bind multiple carbohydrate moieties through the flexible loop sequences within their structures [24]–[26] (Figure 1). GRFT and SVN inhibit HCV in both cell culture and pseudoparticle assays at picomolar and nanomolar concentrations, respectively, and target HCV envelope glycoproteins E1 and E2. In addition, we show that GRFT is active in an albumin (Alb)-urokinase plasminogen activator (uPA)/severe combined immunodeficient (SCID) (Alb-uPA/SCID) chimeric mouse model of HCV infection with up to a 2.5-log reduction of viral titers. The data presented add significantly to recent reports on the anti-HCV activity of GRFT [27] and provide rationale for the continued study of these antiviral proteins as potential therapeutic agents for the treatment of HCV.

## Results

**Figure 2:**
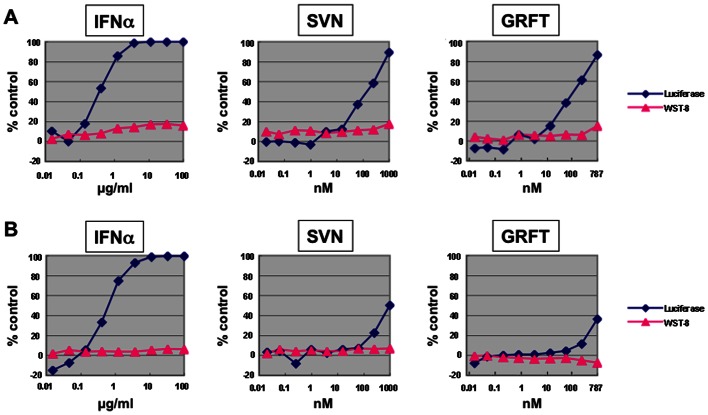
GRFT and SVN are only weakly active in HCV replicon assay. GRFT and SVN were tested in the HCV replicon assay, which does not include viral entry targets. HCV replicon assay was done as described in Materials and Methods using either genotype 1b (A) or 2a (B). The y-axis indicates % inhibition of luciferease activity (light units) (diamond) and cell viability (WST-8 assay) (triangle), respectively. Interferon-a (IFN-α) was used as a positive control.

**Figure 3:**
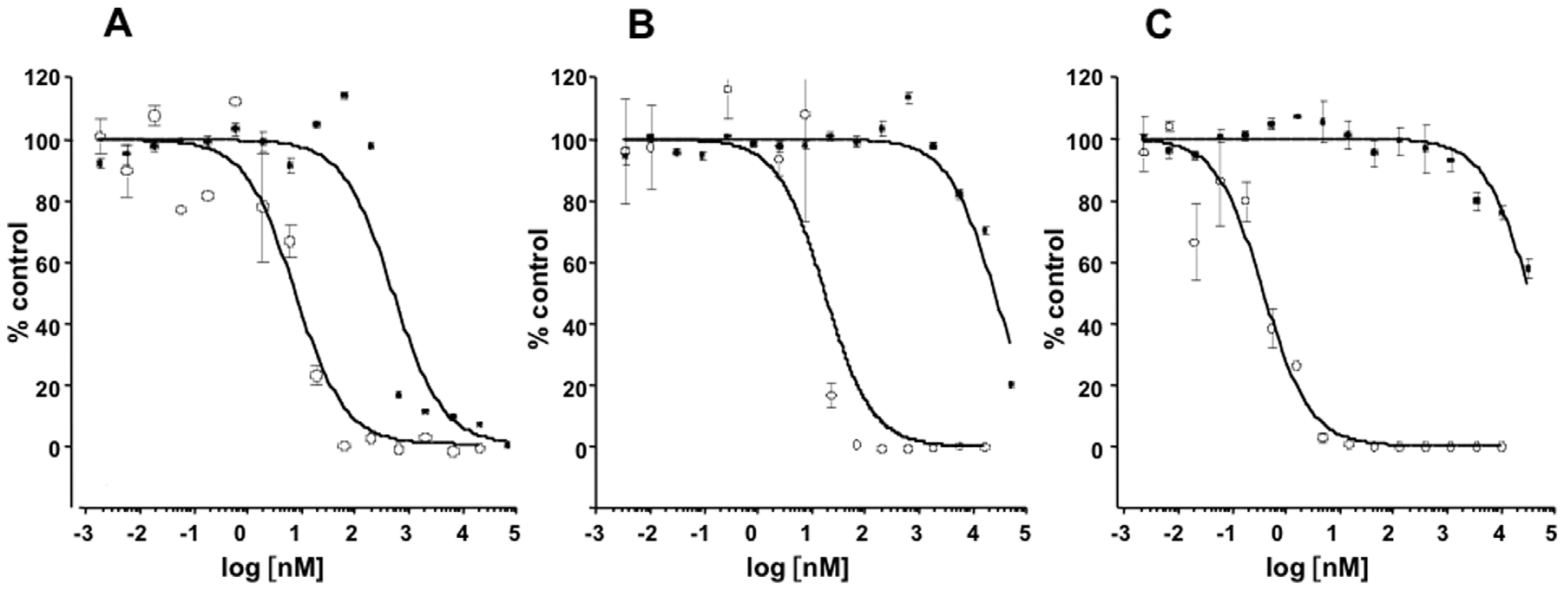
Comparison of anti-HCV activities of GRFT, CV-N and SVN against JFH-1. Anti-HCV activities were evaluated by JFH-1 HCVcc full-replication assay, by measuring HCV core protein output in the culture supernatants of Huh7.5.1 cells 72 hr post-infection. The cytotoxicity was evaluated in parallel using WST-8 assay. The HCV output (open circle) and cell viability (closed square) were plotted as percentage (%) relative to control infections without CV-N (A), SVN (B) or GRFT (C). Compounds were added 15 min prior to viral challenge. Results are expressed as mean and standard deviation from triplicate experiments.

Initial studies on the activity of SVN and GRFT against HCV, performed using a replicon assay system [28], showed minimal activity with 50% effective concentration (EC_50_) values of >200 nM (genotype 1b) and ∼1000 nM (genotype 2a) (Figure 2). In contrast, GRFT and SVN were found to be potently active in a cell-based assay system utilizing the JFH-1 virus and Huh 7.5.1 cells. Both proteins were tested with CV-N as a control for their ability to inhibit viral replication and cell viability. All three proteins were potently active against HCV (Figure 3). SVN was the weakest with an EC_50_ value of 17 nM, GRFT was the most potent with an EC_50_ value of 0.4 nM. GRFT showed low toxicity exhibiting a 50% cytotoxic concentration (CC_50_) = 34 µM and a selectivity index (SI) of 84,000 (Table 1). The significant differences in the results of the JFH-1 cell culture assay and the replicon assay indicated that SVN and GRFT likely worked at a point early in the HCV life cycle, which was not encompassed by the replicon assay.

**Table 1:** Activity of oligomannose-specific lectins from natural product extracts in HCV cell culture assay for inhibition of the type 2a JFH-1 strain of HCV.^a^

Protein	EC_50_ (nM)	CC_50_ (uM)	Selectivity Index (EC_50_/CC_50_)
Cyanovirin-N	7.6	0.39	52
Scytovirin	16.9	23.8	1410
Griffithsin	0.4	33.6	84,000

a CC_50_, 50% cytotoxic concentration; EC_50_, 50% effective concentration.

To expand upon the JFH-1 assay results, additional HCV chimeras encompassing the E1 and E2 proteins from the HCV 2a strain J6 and the HCV 1b strain TH were studied. All three viral strains were sensitive to both GRFT and SVN. Viruses showed variable sensitivity to GRFT and with JFH-1(2a) the most sensitive (EC_50_ = 0.30 nM), TH (1b) intermediate (EC_50_ = 2.2 nM) and J6(2a) the least sensitive (EC_50_ of 14.1 nM). The pattern of sensitivity was similar for SVN although SVN had less potent overall activity (Table 2).

**Table 2:** Inhibitory effect of scytovirin and griffithsin on the replication of JFH-1 and HCVcc chimera.^a^

Strain		JFH-1		J6/JFH-1		TH/JFH-1	
	CC_50_ (µM)	EC_50_(nM)	SI	EC_50_ (nM)	SI	EC_50_(nM)	SI
Scytovirin	4.9	3.2	1,500	50.8	96	57.5	85
Griffithsin	6.4	0.3	21,000	14	460	2.2	2,900

a Inhibitory effect of scytovirin and griffithsin on the replication of JFH-1 and HCVcc chimeras containing structural genes from genotype 2a (J6) or 1b (TH) and nonstructural regions from genotype 2a JFH-1 were quantified by infecting Huh7.5.1 cells.

Anti-HCV activities and cytotoxicity were measured as in Fig. 3. CBP, carbohydrate binding protein; CC_50_, 50% cytotoxic concentration; EC_50_, 50% effective concentration; SI, selectivity index = (EC_50_/CC_50_).

**Figure 4:**
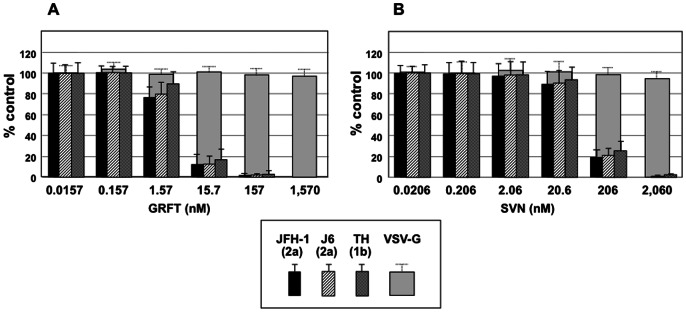
GRFT and SVN inhibit HCVpp entry for different genotypes. Huh7.5.1 cells were incubated for 3 hr with either HCV pseudoparticles(pp) generated with envelope glycoproteins of genotype 2a (JFH-1 and J6 strains) and genotype 1b (TH strain) or VSV-G pp in the presence of increasing amount of GRFT (A) or SVN (B). The inoculum was then removed, and the cells were further incubated. At 2 days post-inoculation, cells were lysed and processed to measure the luciferase activity. The results are presented as percentage of the infectivity relative to infectivity of HCVpp in the absence of carbohydrate binding proteins. Pseudotyped particles produced in the absence of envelope proteins were used as controls, representing <2% of the activity measured for HCVpp. The results are expressed as the mean ± SD in triplicate experiments.

To investigate if GRFT and SVN acted early in the viral lifecycle, they were tested in a HCV pseudoparticle assay which determines the effect of compounds on viral entry [29]. Viral pseudoparticles bearing the envelope proteins of HCV strains JFH-1 and J6 (genotype 2a) and TH (genotype 1b) were again used and compared to control VSV pseudoparticles. GRFT and SVN inhibited all three HCV viruses while having no effect on VSV viral entry. GRFT inhibited HCV pseudoparticle infections at at least one order of magnitude lower concentration that SVN (Figure 4).

**Figure 5:**
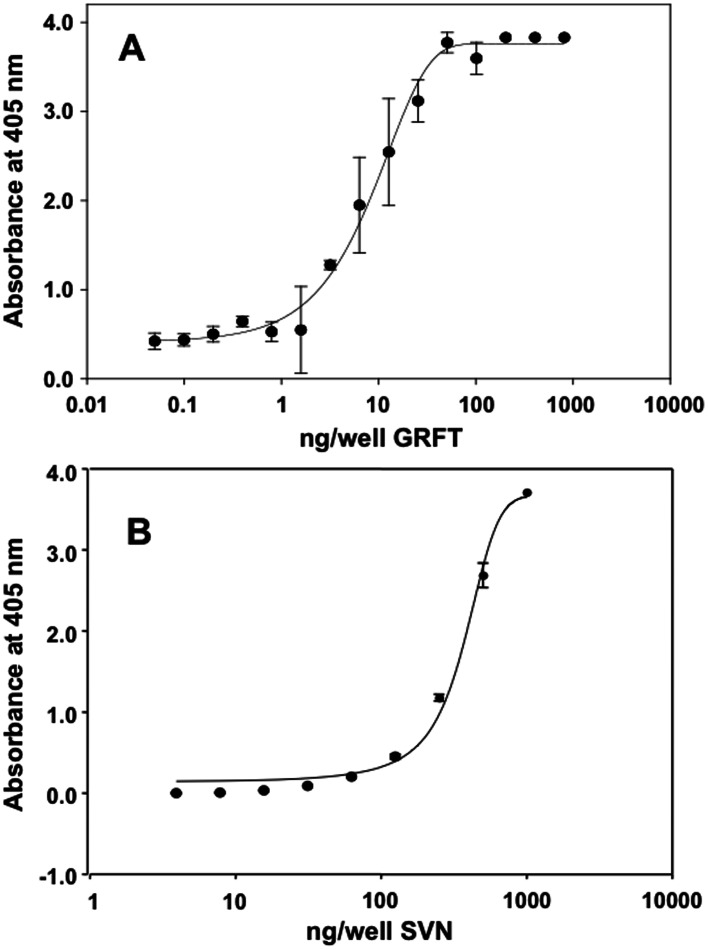
GRFT and SVN bind to HCV E2 glycoprotein. Recombinant glycosylated HCV envelope glycoprotein E2 (strain H77 1a) was bound to 96-well plates and exposed to varying concentrations of both SVN and GRFT. The presence of SVN (A) and GRFT (B) was visualized using rabbit polyclonal anti-GRFT or anti-SVN antibodies as described in the Materials and Methods section. Data points represent the mean of three experiments ± SD.

Following the pseudoparticle assay we attempted to identify which HCV proteins might be binding to both GRFT and SVN by blotting HCV viral lysates and then interrogating the blots with SVN or GRFT. The results indicated that GRFT and SVN both appeared to bind to both HCV E1 and E2 with GRFT also showing binding to many additional bands of protein, while SVN showed a more restricted pattern of binding with E2 the dominant band (MW ∼85–90 kDa) (Figures S1 & S2). We then sought to evaluate the direct binding interaction between HCV E2 and GRFT or SVN. An ELISA assay in which recombinant E2 (Strain H77, 1a) was bound to the plate and then exposed to various concentrations of either GRFT or SVN was used (Figures 5A & B). Both GRFT and SVN show concentration-dependent binding to HCV E2 with the binding curve for GRFT displaying a higher relative binding affinity which mirrors the results seen in the JFH-1 assay system. Similar to previous results with HIV gp120 [23], [24], the binding of GRFT to E2 could be inhibited by the monosaccharides mannose (up to 60% at 100 mM) while the binding to SVN could not (data not shown). This is a result of SVN's more restrictive binding target, an α1-2, α1-2, α1-6 linked tetramannoside [22]. As both SVN and GRFT appeared to bind readily to HCV E2 we further tested their ability to inhibit the subsequent binding of E2 to the cell surface protein CD81. The results of the ELISA experiments clearly showed that neither SVN nor GRFT inhibited the binding of recombinant E2 (Strain H77, 1a) to CD81 (Figure S3).

**Figure 6:**
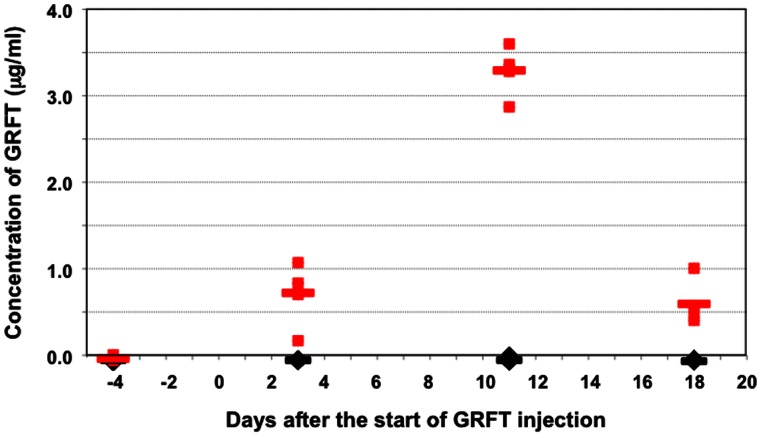
Plasma levels of GRFT following subcutaneous injection into mice. GRFT (20 mg/kg day, Q24) was injected into Alb-uPA/SCID transgenic mice for ten consecutive days. Blood was drawn at the indicated days and plasma was measured for GRFT content by ELISA capture assay as described in the Materials and Methods section. Individual animals are shown as red squares (GRFT-treated, n = 5) or black diamonds (control animals, n = 5). Horizontal bold lines represent the mean values for each group.

Prior to initiating animal efficacy studies, we determined GRFT's bioavailability following sub-cutaneous injection. GRFT (20 mg/kg) was injected into Alb-uPA/SCID mice (n = 5) for 10 days (Q24) and blood samples were taken on days −4, 3, 11, 18 and 25. Quantification of the GRFT present in the mouse plasma was determined using capture ELISA. GRFT was bioavailable following subcutaneous injection, with plasma levels reaching a high of 3.3 µg/mL (∼260 nM) on day 11 and slowly reducing to 0.59 µg/mL (∼47 nM) on day 18 (Figure 6).

Monitoring of health parameters confirmed that GRFT treatment was well tolerated by the animals and resulted in only mild and transient alterations in body weights and morbidity scores in a minority of animals.

**Figure 7:**
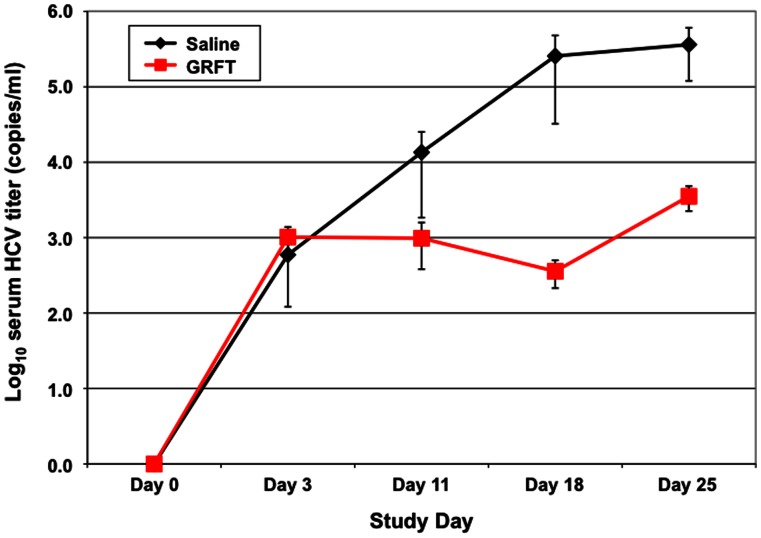
GRFT activity *in vivo* at reducing viral titers after challenge with HCV. Serum HCV titer changes for saline control (black, n = 6) and griffithsin treated (red, n = 6) Alb-uPA/SCID transgenic mice engrafted with human hepatocytes during a 10-dvday treatment regimen. Error bars show the standard error for values at each time point. The minimum detectable limit of the virus load assay is 200 IU/ml.

Following bioavailability and tolerability studies in the Alb-uPA/SCID chimeric mice, we initiated efficacy studies in these same mice after successful engraftment of human hepatocytes. Study animals were split into two groups (n = 6 each) and were dosed (Q24) with either 20 mg/kg/day of GRFT or with saline control. Drug treatment began on day 1 and continued through day 10. Animals were challenged with genotype 1a HCV 8 hr after the first GRFT treatment on Day 1. GRFT treatment was well tolerated and all animals completed the study. Assay of blood samples drawn on days −4 (control), 3, 11, 18 and 25 confirmed that serum hAAT levels remained high and relatively constant for the study animals throughout the study. HCV titers for both GRFT-treated and control animals rose from their pre-infection values of 0 on Day 0 to similar values at day 3 of the study. Thereafter, however, control animal HCV titers continued to rise while GRFT-treated animals showed no increase in titers at day 11 and a reduction in viral titers at day 18 (one week after the final GRFT dose) (Fig. 7). In comparison to control animals, GRFT-treated animals showed reduction of 2.5 log in HCV viral titer on day 18. On day 25, HCV titers in GRFT-treated mice rebounded as would be expected due to the lack of measurable GRFT concentrations in mouse plasma 15 days after the last administered dose of GRFT.

Though the initial *in vivo* study showed efficacy for GRFT in the transgenic mouse model system, the results were not conclusive. To further elaborate on the initial *in vivo* findings, a second study was conducted with several changes. In this study, GRFT was administered on day −1, day 0 and day 1 to allow blood levels to reach therapeutic levels prior to viral challenge, and GRFT (20 mg/kg/day) was administered for a total of 18 days. In addition, GRFT efficacy was compared to IFN- α-treatment (1,350,000 IU/kg/day) in a second study arm while in a third arm animals were treated with both agents to determine if there were any additive effects. As before, all three arms were compared to saline-treated control animals. Four hours after drug treatment on day 1 animals in all groups received ∼2×10^5^ infectious units HCV/dose by i.p. injection. Serum samples were taken on day-4 (control), day 3, 10, 17, 24 and 31 and measured for HCV RNA and hAAT.

**Figure 8:**
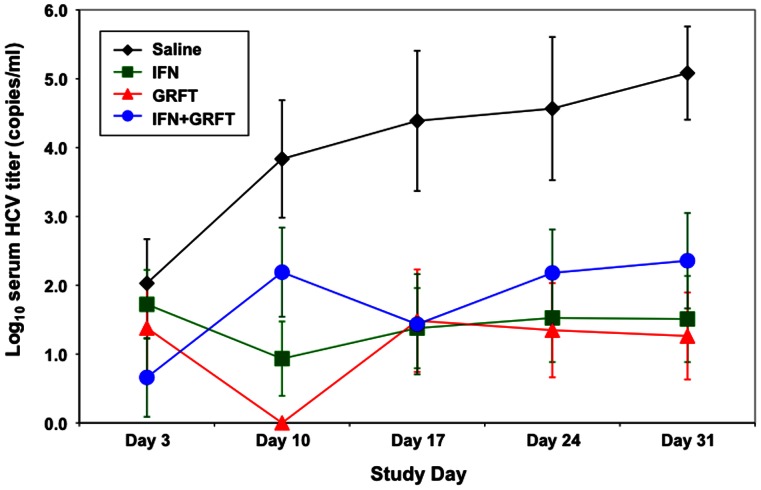
GRFT *in vivo* activity at reducing viral titers prior to challenge with HCV. Serum HCV titer changes for griffithsin treated (blue, n = 6), IFN-α treated (red, n = 6), GRFT+IFNα-treated (green, n = 7) and saline control treated (black, n = 6) Alb-uPA/SCID transgenic mice engrafted with human hepatocytes during an 18-day treatment regimen. On Day 31 the differences in mean log titers between the saline control and both single agent treatment groups were statistically significant: saline vs Interferon p = 0.010; saline vs Griffithsin p = 0.020. The difference in mean log titer between the saline and combination groups was not significant (p = 0.053). Error bars show the standard error for values at each time point. The minimum detectable limit of the virus load assay is 200 IU.

In this second efficacy study the prolonged dosing of GRFT for an additional 8 days appeared to have a deleterious effect on the overall health of test animals with two of seven GRFT–treated mice euthanized after the completion of treatment on day 17 due to excessive weight loss and poor health index scores in addition to one found dead on day one. One animal in the combined GRFT/IFN-α group died on day 11 and two additional mice were euthanized on day 17 in the combination study group. None of the animals in either the saline control or IFN-α-treated groups had to be sacrificed prior to the end of the study. hAAT levels remained relatively constant for all animals in the study except one mouse in the saline control group. Figure 8 shows the time course of the change in average HCV viral titers for all study animals. On Day 10 of the study, none of the animals treated with GRFT had a detectable HCV titer above the assay cutoff of 200 International Units/ml. Five of 6 animals in the saline control group, 2 of 6 in the IFN-α group and 3 of 6 in the GRFT+IFN group had significant viral titers. HCV titers taken on day 17 (one day after the last drug dose on day 16) showed that GRFT-, IFN-α-, and GRFT/IFN-α-treated animals all showed a significant reduction to 2.55–2.64 log HCV International units/ml compared to 4.77 log for control animals. Single factor ANOVA analysis comparing differences in average viral titers between the saline control group and the 3 treatment groups showed that the differences were significant with p values of 0.029 (IFN-α), 0.043 (GRFT) and 0.025 (GRFT+IFN-α). By day 31, 15 days after the last treatment, the average titer for mice given GRFT showed a profound reduction in HCV viral titers to a level of 2.41 compared to 5.08 for control animals. IFN-α-treated mice had a HCV average titer of 2.66 while GRFT/IFN-α-treated mice increased to a level of 2.93 on day 31. These data show that single agent treatment with GRFT was effective in reducing HCV levels and that there was no synergy or additive effects between GRFT and IFN-α in this prevention study.

## Discussion

The need for additional therapeutic agents to treat HCV infections has led to increased attempts to identify both drug targets and candidate drugs that bind to these targets. The envelope proteins of HCV, E1 and E2, are critical for viral infectivity and have not yet been successfully exploited for HCV therapy. Both E1 and E2 are heavily glycosylated with 4–5 conserved *N*-linked glycosylation sites on E1 and 9–11 on E2 [30], [31]. The proper glycosylation of these sites is important both for protein folding and for viral interactions with host receptors [32]. Initially, one agent targeting the glycans on HCV envelope glycoproteins, the lectin cyanovirin-N, was shown to target E1 & E2 and have potent anti-HCV activity in a pseudoparticle HCV assay system [21]. In addition, a recent publication detailed modest activity for the protein GRFT against HCV [27]. We also sought to determine if the high-mannose specific lectins, scytovirin (SVN) and griffithsin (GRFT) with no structural similarity to each other or to CV-N (Figure 1), could show activity against HCV. Initial studies on both proteins using the replicon assay system showed little activity with EC_50_ values of >200 nM (genotype 1b) and ∼1000 nM (genotype 2a) (Figure 2). The fact that there was activity at all in this assay was somewhat surprising as both GRFT and SVN are reported to block viral entry [22], [23] and may indicate that other mechanisms of viral inhibition take place at higher concentrations. Both SVN and GRFT were shown to inhibit HCV infection in the HCV JFH-1 cell culture assay (Figure 3). GRFT inhibited HCV infection at an EC_50_ concentration of 0.4 nM (Table 1) comparing favorably to both SVN (EC_50_ = 17 nM) and CV-N (EC_50_ = 7.6 nM) and showing 30-fold better potency than that recently reported for GRFT [27]. Also impressive was the high *in vitro* selectivity index exhibited by GRFT (SI = 84,000) and SVN (SI >1,400).

To test the hypothesis that GRFT and SVN prevented HCV viral entry we evaluated both proteins in an infectivity assay using pseudoparticles bearing HCV E1 and E2 from JFH-1 (genotype 2a), J6 (genotype 2a) and TH (genotype 1b). GRFT and SVN inhibited all of the HCV pseudoparticles from infecting cells while having no effect on controls (Figure 4A & B). This confirmed that both proteins likely inhibit viral entry. GRFT and SVN were further tested against multiple strains of HCV in the cell culture assay. GRFT was able to inhibit both type 2a (JFH-1 and J6) and type 1b (TH) HCV strains at EC_50_'s ranging from 0.3–14.1 nM while SVN inhibited in the range from 3.2–96 nM (Table 2). The broad-spectrum activity of these proteins against HCV can be explained by their targeting of high-mannose oligosaccharides on either E1 or E2 (Figure 5A & B; S1 & S2), which are common to both genotypes while differences between the results from cell-culture and pseudoparticle assay systems and between individual strains can be attributed to experimental variation and differences in both glycosylation and replication rates between the test systems [33].

Recently, GRFT has been tested in a mouse model of HCV [27]. This small pre-treatment study showed some activity for GRFT with a 1-log reduction in HCV viral titers. GRFT was selected for use in our animal experiments due to its greater potency and higher *in vitro* selectivity index. Bioavailability studies (Figure 6) showed that GRFT was readily distributed following subcutaneous injection and did not cause any deleterious effects on animals after 10 days of dosing (20 mg/kg/day). This result was similar to toxicity results previously reported for both wild type mice and guinea pigs [27]. Whether or not GRFT was immunogenic could be a complicating factor for future development of GRFT. Recently published studies show that GRFT is not immunogenic or activating of immune response, unlike other lectins such as concanavalin A and CV-N [34]. Treatment studies initiated in the Alb-uPA/SCID transgenic mice, showed good efficacy for GRFT in reducing circulating viral titers by ∼2 log (Figure 7) on days 18 and 25. Viral titers increased somewhat between day 18 and 25, possibly due to the sub-therapeutic doses of circulating GRFT two weeks after the last dose was administered on day 11. Our results show that GRFT can be safe and active when given on the same day as an HCV challenge.

To determine if a longer dosing regimen of GRFT could further decrease HCV viral titers, we initiated an 18-day dosing schedule and added a third arm to the study to determine if GRFT activity was additive with IFN–α activity. In addition, GRFT dosing was initiated prior to viral challenge to allow the levels of circulating GRFT to reach better therapeutic concentrations (Figure 6). The results indicated that the longer dosing regimen was not well tolerated by the Alb-uPA/SCID transgenic mice with 6/14 animals in the GRFT-treated groups not surviving past day 24. The increased mortality rate seen in this study may well reflect the sensitivity of the immunodeficient transgenic mice to interventions over a period of several weeks [35]. Indeed, as the level of engraftment for both HCV challenge studies were similar and hAAT values for all animals treated with GRFT remained high for the duration of the study, the toxicity displayed appears to be specific to the inherent fragility of the mouse model and not to toxicity to the implanted human hepatocytes. The longer dosing schedule showed increased activity against HCV in surviving animals with statistically significant mean viral titer reductions of ∼2.7 log by day 31 (p = 0.020) (Figure 8). GRFT+IFN–αtreatment did not show any enhancement relative to treatment with either agent alone. The lack of improvement in outcomes from combination treatment might be due to the fact that both GRFT and IFN-α act to prevent subsequent rounds of infection by HCV; GRFT by blocking viral entry and IFN-α by suppressing the expression of HCV in infected cells [36]. Though the longer treatment regimen showed increased reduction in HCV titers, the reduced survival rate for this treatment regimen was unacceptable. Additional studies to determine the minimum effective *in vivo* dose are planned to determine if a safer dosing regimen can be designed. Furthermore, though in this study only GRFT was tested in the transgenic mouse model system, the *in vitro* activity displayed by SVN recommends it for further evaluation *in vivo* as well.

The ability of the entry inhibitor GRFT to reduce viral titers in an animal model system opens the door for further study of HCV entry inhibitors in general. In addition to further studies with GRFT dosing regimens or SVN, other combinations of these proteins with additional classes of HCV inhibitors, such as the HCV protease inhibitors boceprivir and telaprevir, are merited. Though both of these clinically approved agents show excellent activity in reducing HCV titers, they both have been reported to rapidly generate resistant virus when used as single agents [37], [38]. While GRFT showed somewhat smaller reductions in viral titers than the protease inhibitors in this animal model system, it did not appear to engender resistance. Taken together with the previously published study of GRFT activity against HCV [27], the evidence presented here indicates that GRFT has potential utility. Conceivably, a mechanism of viral entry inhibition could be useful in combination with the protease inhibitors by delaying the onset of resistance to these targeted drugs. Whether GRFT, SVN or other viral entry inhibitors can fulfill this role will require additional studies to determine.

## Materials and Methods

### Reagents

CV-N, SVN and GRFT used in *in vitro* studies were all produced in *E. coli* as previously reported [39]–[41]. GRFT used in animal studies was produced in *Nicotiana benthamiana* as previously reported [42]. Polyclonal antibodies to SVN and GRFT were produced under contract (BioCon, Rockville, MD). Recombinant glycosylated HCV E2 (genotype 1a strain H77) protein was purchased from Immuno Diagnostics Inc.. All other reagents were purchased from Sigma-Aldrich (St. Louis, MO) or Thermo Scientific (Rockford, IL).

### Cells and culture conditions

Huh7.5.1 cells (a gift from Dr. F. Chisari [43]) were maintained in Dulbecco's modified minimal essential medium (DMEM) (Invitrogen, Carlsbad, CA) supplemented with 10% fetal calf serum, 0.1 mM nonessential amino acids, 10 mM HEPES, 100 units/ml penicillin, and 100 µg/ml streptomycin. Replicon cell lines were selected and maintained in 0.5 mg/ml G418 (Geneticin, Invitrogen).

### Cell-culture HCV (HCVcc) full-replication assay

HCVcc were JFH-1 [44] and chimeric viruses containing structural genes from genotype 1b (TH) (Shirakura and Wakita et al., manuscript in preparation) or 2a (J6) and nonstructural regions from genotype 2a JFH-1. Infections with JFH-1 and HCVcc chimeras were quantified by infecting Huh7.5.1 cells with or without test compounds, incubating at 37°C for 3–4 days, and measuring HCV core protein in the culture supernatants using Ortho HCV antigen ELISA kit (Orthoclinical, Tokyo, Japan). Test compounds were added 15 min prior to HCV infection. The cytotoxicity of each compound was evaluated in parallel by WST-8 assay (Dojin, Osaka, Japan).

### HCV replicon assay

Genotype 1b (Con1) and genotype 2a (JFH-1) replicon cells were grown and treated as previously reported [28]. Compounds were added to cells at a 1∶200 dilution, in 96-well assays, 3-fold serial dilutions of test compounds were used.

### HCV pseudoparticle infection assay

Human immunodeficiency virus-1 (HIV-1)-based pseudoparticles containing HCV E1E2 or vesicular stomatitis virus (VSV) glycoprotein G envelope proteins were prepared as described [29]. Pseudotyped viruses were added to Huh7.5.1 cells and incubated for 3 hr at 37°C. GRFT and SVN were added 15 min prior to virus addition. The supernatants were removed, and the cells were incubated at 37°C. At 72 hr post-infection, luciferase activities were measured.

### Western blot of HCV lysates

Whole cell lysates of Huh7.5 cells infected with HCV and uninfected were used: 20 µg protein per lane was used. Samples were resolved in duplicate on two 4–20% Bis-Tris pre-cast gradient SDS-PAGE gels (Bio-Rad, Hercules, CA) and blotted onto polyvinylidine difluoride membranes. Both membranes were blocked with 5% BSA overnight. Membrane 1 was used for detection of HCV E1 and E2 proteins while membrane 2 was used for GRFT and SVN detection. Membrane 1 was incubated overnight (4°C) with mouse anti-E1 HCV antigen monoclonal antibody (1∶50 dilution, Thermo Scientific, Rockford, IL), before being washed three times with PBST and further incubated with goat anti-mouse IgG (H+L), peroxidase conjugated secondary antibody (Thermo Fisher Scientific Inc., Rockford, IL) at a 1∶1000 dilution for 1 hr. After washing with PBST, signals were detected on Hyblot CL autoradiography film (Denville Scientific, Metuchen, NJ) using the SignalFire ECL reagent system (Cell Signaling Technology Inc., Danvers, MA) according to the manufacturer's protocol. Membrane 1 was then stripped to remove all bound antibodies and blocked with 5% blocking buffer for 3 hrs before incubation with anti-gp70(E2) HCV monoclonal antibody (1 µg/ml, ImmunoDiagnostics, Woburn, MA) overnight (4°C). Washing, incubation with secondary antibody and detection were done as previously outlined. For detection of where GRFT binds, membrane 2 was incubated overnight (4°C) with 1 µg/ml GRFT before being washed three times and further incubated with rabbit anti-GRFT polyclonal antibody (1∶200 dilution) overnight (4°C). Membrane 2 was washed as before and incubated with goat anti-rabbit IgG (H+L), peroxidase conjugated secondary antibody (Thermo Fisher Scientific Inc., Rockford, IL) at a 1∶1000 dilution for 1 hr. Washing and detection was done as before. For detection of SVN binding, membrane 2 was stripped and blocked as previously outlined and incubated with 1 µg/ml SVN overnight (4°C) before being washed three times and further incubated with rabbit anti-SVN polyclonal antibody (1∶5000 dilution) overnight (4°C). Washing, incubation with secondary antibody and detection were done as previously outlined.

### ELISA for GRFT and SVN binding to HCV E2

100 µl of a 1 µg/ml solution of recombinant HCV E2 (Strain H77, 1a, Immuno Diagnostics Inc.) in sterile filtered PBS was added to each well of a protein-binding 96-well plate (Nunc, Maxisorp). ELISA was then performed with both GRFT and SVN as previously reported either in the presence of increasing concentrations of mannose (0–100 mM) [23], [24].

### Effect of GRFT and SVN on HCV E2 binding to CD81

To test the effect of pretreatment with GRFT or SVN on E2 binding to the cell surface protein CD81, 100 ng/well of recombinant HCV 70 kDa E2 envelope protein (Immuno Diagnostics, Woburn, MA) was bound to high-binding 96-well Greiner plates (VWR, Bridgeport, NJ) at 4°C overnight. The wells were emptied and then blocked with 200 µl/well of 3% BSA (Sigma, St. Louis, MO) overnight before being washed three times with 200 µl/well PBST. Serial dilutions of GRFT or SVN were added (100 µl/well) to the test wells while 100 µl/well PBST was added to the control wells; incubation done at room temperature for 1 hr 30 min. Wells were washed as before and serial dilutions of human recombinant CD81 (25–127) protein (Abnova, Taipei, Taiwan) were added to both test and control wells; incubation done for 1 hr 30 min. Wells were washed as before and 100 µl/well monoclonal mouse anti-CD81 (25–127) antibody (1∶1000, Abnova, Taipei, Taiwan) was added and incubated for 1 hr30 min at room temperature, followed by three washes with PBST. Goat anti-mouse IgG (H+L), peroxidase conjugated secondary antibody (Thermo Fisher Scientific Inc., Rockford, IL) was added to each well (100 µl/well of a 1∶1000 dilution) and further incubated at room temperature for 1 hr, followed by three washes with PBST. The TMB 2-component Microwell peroxidase substrate kit (KPL Inc., Gaithersburg, MD) was used for peroxidase detection: 100 µl/well added, incubation for 30 min (GRFT test plate) or 100 min (SVN test plate) before reaction was stopped with 100 µl/well 0.5M HCl. Absorbance read at 450 nm.

### Bioavailability and tolerability study on subcutaneous injection of GRFT into Alb-uPA/SCID mice

Ten albumin-urokinase plasminogen activator/severe combined immunodeficient (Alb-uPA/SCID) mice with low engraftment of human liver cells (human α1-antitrypsin (hAAT) values less than 20 at 6 weeks) were used. Individual body weights were recorded prior to treatment and daily during the study. Animals were evaluated daily for morbidity and mortality. Study animals were allocated into 2 groups of 5 animals. GRFT or saline solutions were administered daily by subcutaneous injection. Dosing volume was 2.0 mL/kg for both groups. Blood was collected *via* the central tail artery from all animals, allowed to clot and serum removed. Dosing began on Day 1 and continued daily up to and including Day 10. Blood samples were collected on Days 3, 11, 18 and 25. Serum samples were stored at −80°C prior to assay. GRFT levels in mouse serum were quantified by ELISA on gp120 coated plates as previously reported [39] using a standard curve of GRFT spiked mouse serum.

### Ten-day treatment schedule with GRFT and challenge with HCV

Twelve Alb-uPA/SCID mice with high engraftment of human liver cells (hAAT values greater than 100 ng/ml at baseline and not previously infected with HCV) were allocated to this study. Animals were evaluated as before except that study animals were allocated into 2 groups of 6 animals.

Treatment began on Day 1 and continued daily up to and including Day 10. Animals were challenged with HCV 8 hours after the first drug dose on Day 1 by intra-peritoneal injection of 100 µl of a standard patient serum laden with genotype 1a HCV (2.0×10^5^ IU). Blood samples were collected on Days 3, 11, 18 and 25 and the sera separated. Serum samples were stored at −80°C until used for measurement of hAAT and HCV titers. The minimum detectable limit of the virus load assay is 200IU.

### 18-day treatment schedule with GRFT and IFN-α and challenge with HCV

Twenty-five Alb-uPA/SCID mice were allocated to this study. Animals not previously infected with HCV were evaluated as before and allocated into the 4 different groups. Group 1 animals (n = 6) received saline control, Group 2 animals (n = 6) received INF-α (1350 IU/g), Group 3 animals (n = 6) received GRFT (20 mg/kg) and Group 4 animals (n = 7) received a combination of GRFT and INF-α.

Baseline blood draws were obtained on Day −4 and daily GRFT administered on Day −1 and Day 0. On Day 1 of the study, animals were treated with GRFT, challenged 4 hours later by the intra-peritoneal injection of 100 ul of the HCV genotype 1a inoculum (2.0×10^5^ IU) and then dosed with INF-α (for the Group 2 INF-α group and the Group 4 combination group). Dosing with GRFT, INF-α or the combination of the two continued daily up to Day 16. Blood samples were collected on Days 3, 10, 17, 24 and 31. Serum samples were prepared and stored at −80°C until assayed for hAAT and HCV titers. The minimum detectable limit of the virus load assay is 200IU.

All animals used in the studies above received humane care according to the criteria outlined in the “Guide for Care and Use of Laboratory Animals”, National Academy of Sciences.

## Supporting Information

Figure S1 **Western blot analysis of GRFT interaction with HCV infected and uninfected Huh7.5 cells.** Lane 1: molecular weight marker (masses shown in kilodaltons on the right); lanes 2–9: whole cell lysates from HCV infected Huh7.5 cells (20 µg protein/lane); lanes 10: whole cell lysate from uninfected Huh7.5 cells (20 µg protein/lane). After overnight Incubation with GRFT (1 µg/ml), bound GRFT was detected with rabbit anti-GRFT polyclonal antibodies at a 1∶200 dilution.
(TIFF)

Figure S2 **Western blot analysis of SVN interaction with HCV infected and uninfected Huh7.5 cells.** Lane 1: molecular weight marker (masses shown in kilodaltons on the right); lanes 2–9: whole cell lysates from HCV infected Huh7.5 cells (20 µg protein/lane); lane 10: whole cell lysate from uninfected Huh7.5 cells (20 µg protein/lane):. After overnight Incubation with SVN (1 µg/ml), bound SVN was detected with rabbit anti-SVN polyclonal antibodies at a 1∶5000 dilution.
(TIFF)

Figure S3 **ELISA study on the effect of GRFT (A) or SVN (B) on HCV E2 binding to CD81.** Plate-bound E2 protein at 100 ng/well, was pre-treated with (▪) or without (•) GRFT or SVN (100 ng/well) for 1 hr30 minutes, before serial dilutions of CD81 were added. Mouse anti-CD81 monoclonal antibodies were used to detect the bound CD81 as indicated by absorbance readings. Points are averages of triplicate samples (corrected for the blocking agent background values).
(TIFF)
