# Supplementary figures and images for: Antiviral Lectins from Red and Blue-Green Algae Show Potent In Vitro and In Vivo Activity against Hepatitis C Virus
Source: PLoS One. 2013 May 21;8(5):e64449. doi: 10.1371/journal.pone.0064449 (PMC3660260; doi:10.1371/journal.pone.0064449)

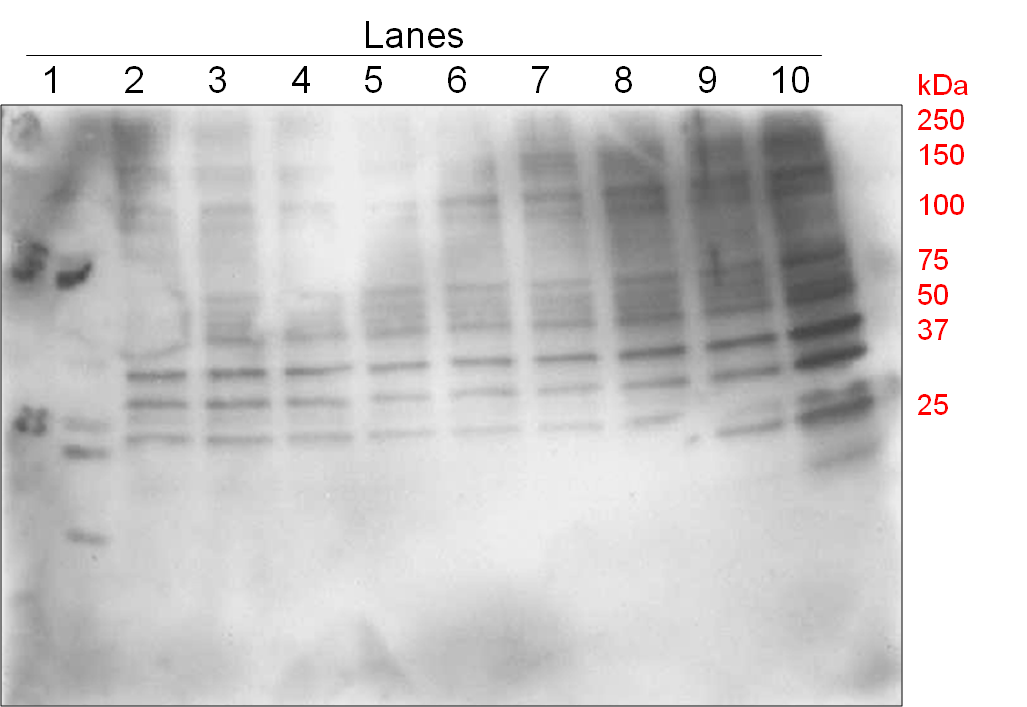

Supplement: Figure S1 — Western blot analysis of GRFT interaction with HCV infected and uninfected Huh7.5 cells. Lane 1: molecular weight marker (masses shown in kilodaltons on the right); lanes 2–9: whole cell lysates from HCV infected Huh7.5 cells (20 µg protein/lane); lanes 10: whole cell lysate from uninfected Huh7.5 cells (20 µg protein/lane). After overnight Incubation with GRFT (1 µg/ml), bound GRFT was detected with rabbit anti-GRFT polyclonal antibodies at a 1∶200 dilution. (TIFF) [file pone.0064449.s001.tiff]

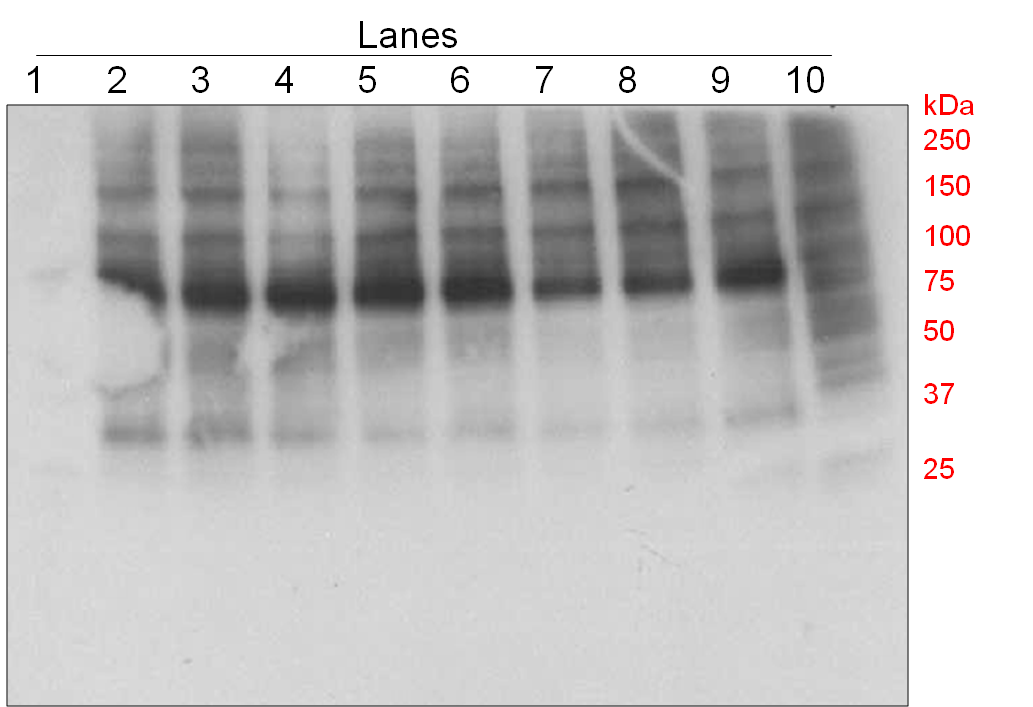

Supplement: Figure S2 — Western blot analysis of SVN interaction with HCV infected and uninfected Huh7.5 cells. Lane 1: molecular weight marker (masses shown in kilodaltons on the right); lanes 2–9: whole cell lysates from HCV infected Huh7.5 cells (20 µg protein/lane); lane 10: whole cell lysate from uninfected Huh7.5 cells (20 µg protein/lane):. After overnight Incubation with SVN (1 µg/ml), bound SVN was detected with rabbit anti-SVN polyclonal antibodies at a 1∶5000 dilution. (TIFF) [file pone.0064449.s002.tiff]

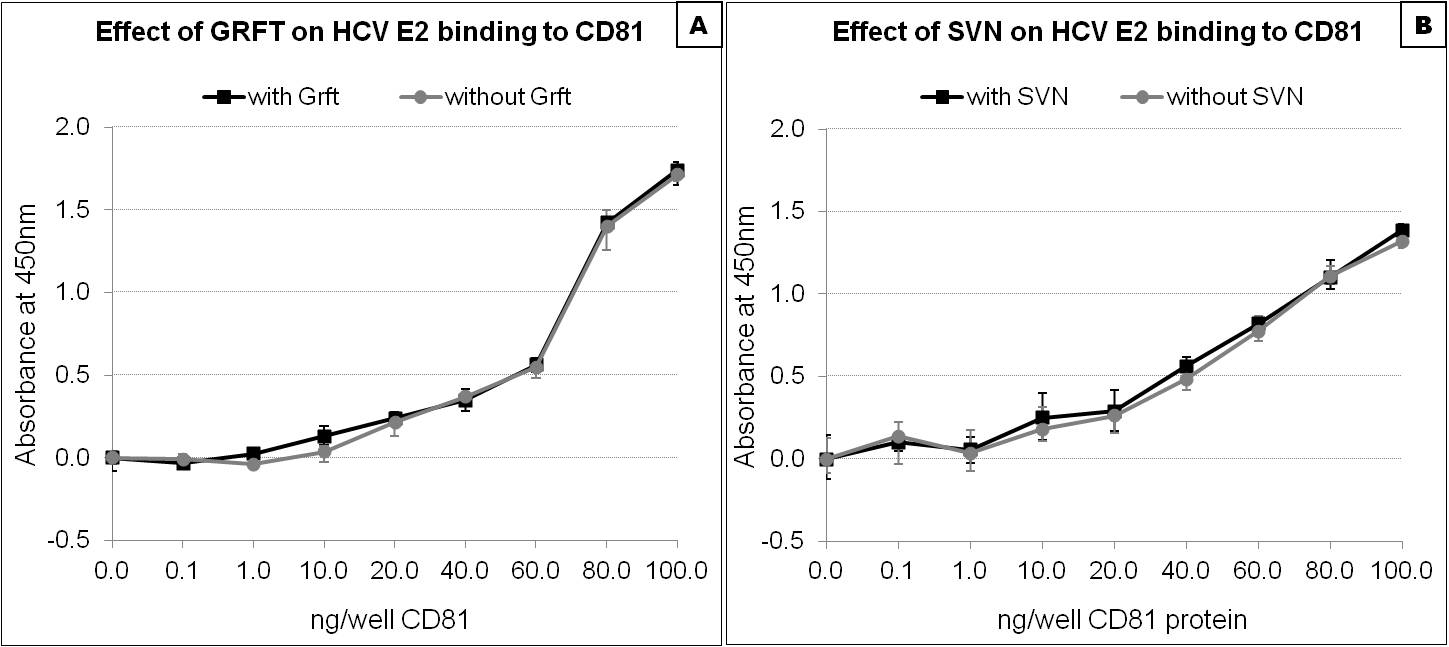

Supplement: Figure S3 — ELISA study on the effect of GRFT (A) or SVN (B) on HCV E2 binding to CD81. Plate-bound E2 protein at 100 ng/well, was pre-treated with (▪) or without (•) GRFT or SVN (100 ng/well) for 1 hr30 minutes, before serial dilutions of CD81 were added. Mouse anti-CD81 monoclonal antibodies were used to detect the bound CD81 as indicated by absorbance readings. Points are averages of triplicate samples (corrected for the blocking agent background values). (TIFF) [file pone.0064449.s003.tiff]
